# Transforming dental education: interactive and student-centered learning with team-based learning in the undergraduate program

**DOI:** 10.3389/fmed.2025.1579237

**Published:** 2025-06-03

**Authors:** Teun J. de Vries, Katie L. Crouwel, Erica Vogelzang, A. Isa Levert, Egbert Neels, Amber de Wilde, Pepijn Koopman, Denise E. van Diermen, Geerling E. J. Langenbach, E. Etienne Verheijck

**Affiliations:** ^1^Department of Periodontology, Academic Centre for Dentistry Amsterdam, University of Amsterdam and Vrije Universiteit, Amsterdam, Netherlands; ^2^Department of Education, Academic Centre for Dentistry Amsterdam, University of Amsterdam and Vrije Universiteit, Amsterdam, Netherlands; ^3^Reflect Academy, Driebergen, Netherlands; ^4^Department of Information and Communication Technology in Education, Academic Centre for Dentistry Amsterdam, University of Amsterdam and Vrije Universiteit, Amsterdam, Netherlands; ^5^Department of Oral Medicine and Orofacial Anatomy, Academic Centre for Dentistry Amsterdam, University of Amsterdam and Vrije Universiteit, Amsterdam, Netherlands; ^6^Department of Oral Pain and Disfunction, Academic Centre for Dentistry Amsterdam, University of Amsterdam and Vrije Universiteit, Amsterdam, Netherlands

**Keywords:** dentistry, team-based learning, active teaching and learning, evaluation, implementation

## Abstract

World-wide, educational curricula have made a transition toward (inter)active student-centered learning and teaching. To incorporate consistency within a curricular reform, it is important to make choices that are applied to all courses. Here we describe the implementation of team-based learning (TBL), an effective educational approach for activated learning at a dental school. TBL stimulates students to participate actively in their own learning process. This team-oriented method fosters problem solving, critical academic reasoning, clinical decision-making and communication skills among students, already early in their educational career. In the first year of the undergraduate program, TBL was introduced as a mandatory component, constituting 10% of the teaching activities and overall grade. To facilitate this transition, a dedicated team of teachers and educationalists (the TBL team) was formed to prepare the transition. The initial step involved establishing a TBL course and conducting training sessions for faculty to familiarize them with this new teaching methodology. Teachers received constructive feed-back on their own TBL application session. Due to the Covid-19 pandemic, TBL was introduced as an online variant, requiring close collaboration with IT-services. Halfway through the academic year, the implementation was evaluated through separate panel discussions with students and teachers separately. Overall, TBL was perceived favorably by both staff and students. Students appreciated the team work and noted that TBL added value to their learning process. This was also the outcome of the end of the academic years’ student survey on TBL, where especially questions on collaborative teamwork scored 4.22 on average on a 1–5 Likert’s Scale. TBL was inspiring for teachers, the student teams of TBL provided a safe environment for students to voice their thoughts. The activating nature of TBL was recognized as beneficial, though it requires continuous effort and motivation from instructors. Coaching and guiding were perceived as highly effective instructional methods. Some teachers acknowledged the challenge of transitioning from a traditional “one-person” show approach to a more collaborative teaching style. Both evaluations facilitated further refinement of the TBL approach. Particularly, during the social intercourse-deprived Covid-19 era, the fixed-groups format of TBL helped students to experience a sense of belonging.

## 1 Introduction

### 1.1 Background and rationale for the educational activity innovation

In the rapidly evolving landscape of education, continuous innovation in curriculum design, instructional strategies, and pedagogical frameworks is essential. While lectures are effective for information delivery, they are less suited for promoting higher-order thinking (1). Because of their proven effectiveness (2, 3), active teaching and learning methods have become an integrated part of curricula in higher education. Pedagogical frameworks like flipped classroom (4, 5) and team-based learning (6), emphasize student engagement and the cultivation of self-directed learning skills.

The Academic Centre for Dentistry Amsterdam (ACTA) has taken significant strides in reforming its undergraduate program to incorporate active teaching and learning methodologies, with a particular emphasis on team-based learning (TBL). For the curricular reform at ACTA, it was decided to implement TBL as the principal method and was introduced for 10% of teaching and learning time for all courses of the first year of the bachelor program. This has been gradually expanded to all courses of all years of the bachelor program. The choice for TBL was partially based on good experience at the neighboring Amsterdam University Medical Centre (7, 8). TBL has grown exponentially in the health profession education between 2011 and 2016 (9) and is therefore a choice of activated teaching and learning that has shown its value. Effectiveness of TBL has also been studied in the field of dentistry. A meta-analysis of active learning strategies among dentistry undergraduates across 93 studies, concluded that active learning not only improves satisfaction, but also enhances knowledge acquisition, outperforming traditional teaching methods (10). A recent scoping review on TBL at dentistry faculties confirmed these outcomes. When compared with traditional teaching, student satisfaction and performance improved after implementing TBL (11).

For successful implementation of teaching and learning methods from scratch, it is desirable to implement one principal form of activating teaching and learning, since both teachers and students need time to be instructed and to be familiarized with a new way of teaching and learning. Proper preparation is therefore instrumental. Many curricular innovations fail due to too little guidance during these changes (12). For a successful implementation, it is important to introduce a step-by-step plan. First teachers and students have to be trained in the new method. Secondly, during the initial year, it is important to organize panel discussions with all users, both teachers and students. Finally, the incorporation of a new educational method should be evaluated for all courses and as an umbrella at the end of the year for all TBLs. For the latter, the entire first year filled out a questionnaire on the various educational and organizational aspects of TBL. Here we describe which preparations were made, how they were implemented in the courses of the first year and how it was perceived by both students and teachers.

## 2 Pedagogical framework(s), pedagogical principles, competencies/standards underlying the educational activity

### 2.1 What is team-based learning?

Team-based learning contains an individual study phase that is followed by the so-called readiness assurance test (RAT) containing 15–25 multiple choice questions. Thus, knowledge at the cognitive levels of taxonomy of Bloom, understanding and remembering (13); modified by (1) are tested. This test is first done individually (iRAT), then taken again, now with the pre-formed team, the team-RAT or tRAT. Answering the questions again as a team contributes to the learning process. After this, readiness for the application session is assured. In other words, students are prepared to answer more complex questions together in their pre-formed teams. This takes place during the application phase of TBL, which lasts about 2 h during which approximately three more complex multiple-choice questions can be answered. Importantly, the various TBL-teams work together and tackle the same problems. Answers are given simultaneously after the teams have been given time for deliberation. For the design of complex questions during the application phase, it is important that all possible answers can be defended. Thus, TBL prepares students for formulating academic arguments and is thus suitable for training academic reasoning and clinical decision making. Throughout the application phase of TBL, students are in the lead. The teacher adapts to a role of coach, occasionally adding expert knowledge (6, 14, 15).

## 3 Learning environment (setting, students, faculty); learning objectives; pedagogical format

Academic Centre for Dentistry Amsterdam enrolls approximately 128 students annually, making it the largest dental school in Netherlands and one of the largest in Europe. The learning environment is designed to facilitate active engagement, with innovations such as the thematic organization of the digital learning environment, Canvas, and the incorporation of small group tutorials and seminars. The undergraduate program at ACTA gradually progresses from theoretical foundations to the acquisition of pre-clinical skills, preparing students for clinical practice in the second and third year. The emphasis in the 3 years Master’s program is on deepening fundamental knowledge and on acquiring clinical skills.

For the implementation of TBL in the Bachelor, the following sequential steps were undertaken.

### 3.1 Preliminary engagement with faculty

Approximately 9 months prior to the scheduled implementation of TBL, a workshop aimed at introducing the methodology to faculty members was conducted (DvD). This workshop, attended by 15 ACTA educators, comprehensively covered all stages of TBL, including the preparation phase, individual Readiness Assurance Tests (iRAT) iRAT, team Readiness Assurance Tests (tRAT) and the application phase).

### 3.2 Formation of the TBL implementation team

To ensure the effective implementation of TBL, a dedicated team, the TBL team, was established 6 months prior to introduction in September 2020. This interdisciplinary team was comprised of teachers, educational specialists, and experts in higher educational management (including TdV, KC, EV, IL, EN, and later AdW). Since the team did not contain TBL experts, TBL experts at the Amsterdam Medical Centre (AMC) were consulted nearly at a weekly basis. In the past, EEV was as director of the bachelor program as expert involved in implementing TBL at AMC and was consulted on a regular basis. For adding TBL expertise in the team, one of the team members (EV) followed the fundamentals series of the Team-based learning collaborative (TBLC). The team’s primary objective was to develop and design the necessary resources for both faculty and students. The following were developed by the TBL team:

#### 3.2.1 Development of a teacher training program

**FIGURE 1:**
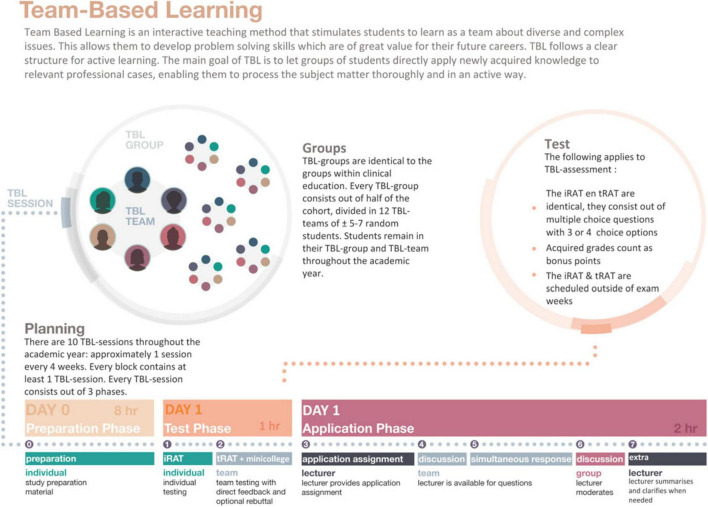
Infographic on team-based learning designed especially for Academic Centre for Dentistry Amsterdam (ACTA) to instruct both teachers and students.

A comprehensive digital course was developed on the Canvas learning platform to familiarize faculty with TBL within approximately one hour. This course featured online video clips, an infographic designed for ACTA’s TBL framework (Figure 1), a video message from the Director of Education (EEV) emphasizing the importance of TBL, and a quiz and examples of application session questions. Following this self-paced course, a 1 h online training session was conducted via Zoom to reinforce key concepts and answer any faculty questions.

### 3.3 Student instruction and preparation

A training module was also developed for first-year students to acquaint them with TBL. This module was delivered through a Zoom lecture and included an instructional video, a breakdown of the TBL process using the previously mentioned infographic (Figure 1), and a rehearsal of an application question. Thus, each step of TBL was explained separately. Team captains were subsequently required to defend their team’s answers, fostering early engagement with TBL’s collaborative learning process.

### 3.4 Course-specific guidance for faculty

For each course incorporating TBL, two advisory sessions were scheduled. The first session, conducted 6–8 weeks before the TBL session, aimed to clarify the TBL framework and assist the member of faculty with the design of the application session. A follow-up session, held 2–4 weeks before the session, provided feedback on the teaching materials developed by the faculty, ensuring alignment with TBL principles. For onboarding for the heads of departments, a session on TBL was organized right after the implementation phase. The RAT questions were typical for 100 level courses, basically reproducing knowledge. These were checked by at least one other teacher of the same TBL. In later years, all questions were handed in beforehand to a specific test expert who provided feedback on the questions.

### 3.5 Integration of TBL-specific evaluation metrics

In collaboration with the Vrije Universiteit Amsterdam, ACTA adapted its course evaluation protocols by including TBL-specific questions. These questions, presented on a Likert scale, assessed students’ perceptions of TBL broadening their understanding, the effectiveness of the TBL instructor as a coach, the overall value of TBL, and the benefit of group work.

### 3.6 Preparing the infrastructure for TBL at ACTA

The global Covid-19 pandemic necessitated the transition to online education across Dutch Universities in the summer of 2020. Consequently, the TBL framework was adapted for online delivery, requiring close collaboration with the ICT support group (PK). Each Canvas course was equipped with links to Zoom meetings, and the RAT phases were conducted using TestVision (an online summative assessment tool) with online proctoring. The tRAT was conducted in Zoom using separate breakout-rooms per team.

The onsite version of TBL makes use of scratch cards, where answer choices are made both for the iRAT and the tRAT, where for the latter, points were deducted for the number of attempts. The implemented online version used the same principles as the scratch card method. For the tRAT, one student filled-in the team choices per question on behalf of the whole group. Results were registered in TestVision.

The cohort was divided into 24 teams of six students, with each application session conducted in four groups of six teams. Each session addressed three application questions, with teams submitting their answers simultaneously via Zoom’s chat function.

Points for iRAT (0.7 grade points on a ten point scale) and tRAT (0.3 grade points) could be earned per course, provided that all TBL-phases were followed, including the application phase of TBL. Attendance at the application phase was established by checking the Zoom meeting attendance reports.

### 3.7 Monitoring and evaluation: the ALERT group

To ensure the continuous monitoring and evaluation of TBL implementation, team TBL participated in the Activated Learning and Teaching (ALERT) group, which convened monthly. This interdisciplinary team provided a platform for discussing the progress of TBL adoption, addressing any challenges, and initiating corrective actions as necessary. The ALERT group furthermore consisted of the manager of the Teaching and Learning Center of ACTA, teaching staff, a member of the ICT support team, and student members. Any points of attention regarding TBL were brought forward to the educational directors of the faculty.

## 4 Results to date/assessment (processes and tools; data planned or already gathered)

### 4.1 Implementation of TBL across courses

**FIGURE 2:**
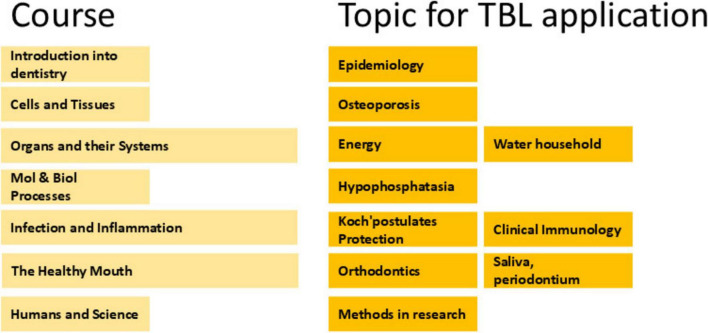
Overview of the courses of bachelor-1 and the corresponding team-based learning (TBL) sessions.

Team-based learning was integrated into all seven courses within the first year undergraduate program. Without exception, TBL became a structural component of every course. In larger 7–8 weeks courses, two TBL sessions were conducted, while shorter 4 weeks courses incorporated one TBL session. The TBL teams stayed intact for all ten TBL sessions, while the role of spokesperson during the application phase rotated, ensuring each team member had the opportunity to practice leadership. Application sessions were organized as thematic components within the courses (Figure 2). The thematic components were chosen by the teachers, often from different departments of ACTA, and were each asked to align to a common theme (16). For the preparatory phase of the day before the TBL day, students were often instructed to study the course thus far, approximately half-way the course. The online format of the TBL application sessions provided a unique opportunity for feedback to the teachers that led the TBL. Each TBL application session was attended by at least one member of Team TBL, who used a feedback form to evaluate organizational aspects, such as transitions between general assembly and breakout rooms, as well as the role of the instructor as facilitator. Following each TBL application session, a brief oral feedback moment with the TBL instructor took place.

During the year of implementation, TBL was monitored in four ways: by mid-year student panels (5.2), mid-year teacher panels (5.3), with TBL specific questions in the regular course evaluations (5.4) and with a TBL student questionnaire (5.5).

### 4.2 Mid-year evaluation: student panel

Team TBL organized a panel discussion for the initial fifteen teaches, of whom eleven attended focusing on four key themes. Participants were year class representatives, students council members and volunteers, all from the first year. A summary of the transcript, including representative quotes is provided below.

On the educational format, students replied that they found the TBL format to be well-structured, particularly the use of breakout rooms. However, some students experienced confusion during the application phase, as the relevant correct answers were occasionally unclear. Apparently, students had to acquaint themselves with the new method where various options can be chosen and argued. The opportunity for discussion was highly valued, with students noting that it was particularly beneficial for exam preparation. Collaborative discussions were described as enjoyable, with differing opinions adding value. The online format was also positively appreciated.

Typical for TBL is that the teacher primarily functions as moderator. Students appreciated additional explanations and summaries provided by the teacher. One teacher was noted for the sense of humor and encouragement, which was positively received. Students suggested that summarization at the end of sessions, by the teacher, would be beneficial.

A hallmark of TBL is collaborative group work. Panel members enjoyed working in fixed teams, which allowed them to become familiar with each other’s strengths and weaknesses. This familiarity facilitated open communication during team discussions, during which students learned from each other’s perspectives. Students observed that long-term team membership enhanced collaboration, with introverted members becoming more vocal and engaged over time. They reported that well-established teams functioned more efficiently, just as a well-oiled machine.

Some valuable student quotes from the panel discussion are listed here. “*You start to feel at ease with each other and this makes you comfortable to say what you want to in the team discussions. The threshold for giving your opinion would be higher if you were always in a newly formed team.*” Almost everyone experienced growth in collaborating with each other. “*The longer you are a team together, the better you know each other and the better you know each other’s qualities. This way you learn to complement each other.*” A student noticed that over time, fellow students who are more introverted become more prominent in the team. They feel comfortable.

“*I noticed that we can solve issues in a team more quickly. It’s faster and easier, like a well-oiled machine.*”

Regarding the positive aspect of TBL, students responded as follows.

- “*I have learned to work together and experience that when I explain the material to someone else, I deal with it differently and more actively. This makes me incorporate the material better.*”
- “*When you actively deal with the learning material and have already taken a test in between, the material will be retained better. You remember things better. You use more senses.*”
- “*When you explain an answer to a question, or have it explained by another student, it will stick better.*”
- “*The tRAT has a valuable function. When assessing the same questions, but now in a team, you immediately see your mistakes.*”

### 4.3 Mid-year evaluation: teacher panel

A panel discussion with eleven teachers was organized by Team TBL, focusing on four themes. A summary of the transcript, including representative quotes, is provided below.

On their experience with the novel educational method of TBL, teachers responded as follows. Several teachers reported that TBL was enjoyable, albeit that it required intensive preparation. The creation of RAT questions and application sessions was perceived as demanding. “*It has given me a lot of inspiration, also for my other teaching activities. I find it very useful that you activate students by having them study the material in advance. It also helps to take a test together.*” TBL was also praised for its ability to engage students and stimulate discussion. “*I think it is a very enjoyable form of education. I can imagine that it is nice for students, too. I liked that I could also contribute bits to the discussion myself, depending on which direction it went. There was more interaction than during a lecture. I found it intensive, two TBL application sessions in a row, with just your screen ahead of you for hours. However, that is an organizational thing, not substantial. Intensive to guide the content and the process. It does ask something of you.*”

Teachers valued the interactive nature of TBL, which encouraged students to deeply engage with the material. The small group discussions created a safer environment for students to voice their thoughts, reducing fear of making mistakes compared to large group settings. However, some challenges were noted, such as the constraints of four-choice question formats and occasional passive participation by students. Teachers observed that students appeared better prepared for other teaching sessions and demonstrated improved performance on insight questions. The activating nature of TBL was recognized as beneficial, though it requires continuous effort and motivation from instructors. “*The biggest added value is that they discuss in small groups, in a safe environment. In a large group they often do not dare to contribute, they are afraid of making mistakes. I think it is a disadvantage that everything has to fit into a four-choice straitjacket, and also a disadvantage that students lean backward.” “We have been sending information for years, and for the first time since 1994 I had the feeling that most of them had prepared, in the breakout rooms they were working on the material, and I saw that reflected in the test results: insight questions have been answered much better.*”

In the context of TBL, instructors are encouraged to step back from directly providing content, instead focusing on guiding students to discover the correct answers collaboratively. The possible answers are revealed at the end of the session, supplemented with additional substantive information if necessary. Peer feedback among colleagues was a valuable component of this process, and the feedback provided by team TBL was instrumental in refining subsequent sessions. One teacher noted: “*It is critical that all TBL sessions are conducted uniformly to ensure consistency for the student. Deviating from this structure undermines the learning process.*” Another teacher emphasized the importance of small-scale, active learning highlighting that TBL not only fosters engagement but also enhances collaboration. “*Coaching and guiding are highly effective instructional methods.*” a teacher remarked. However, some teachers acknowledged the challenge of transitioning from a traditional “one-person” show approach to a more collaborative teaching style. “*This requires a fundamental shift in our teaching methodology*” one teacher noted.

“*I think it is important that all TBL sessions are unambiguous and carried out in the same way. It is important for the students that there is a consistent line. You should not set this up in your own way*.” “*It is about activating learning in a small-scale setting. TBL has the advantage that it also stimulates collaboration. Coaching, guiding, these are very strong working methods.*” “*We struggle as teachers, because we often conduct a one-man-show. That doesn’t really work. In any case, as teachers we are now forced to approach it in a different way.*”

The online implementation of TBL presented certain challenges, primarily due to the lack of a dedicated TBL space. Nevertheless, breakout rooms were considered an excellent alternative under the circumstance of the global COVID-19 pandemic. One teacher commented, “*While TBL is more enjoyable in person, the online format effectively encourages participation from students who are typically less vocal. Interestingly, these quieter students often excel academically.*” The online environment was perceived as more efficient, although the lack of on-site experience was noted. In a physical setting, visual contact facilitates smoother transitions within the team, an aspect somewhat diminished in the digital format. However, visiting breakout rooms online partially mitigated this issue. Support from the ICTO (Information and Communication Technology in Education) team was considered essential for the successful execution of online TBL sessions.

The support of Team TBL was indispensible, particularly in preparing TBL sessions from scratch. One teacher reflected: “*I appreciated the opportunity to collaborate with colleagues, which made the experience less daunting. The guidance provided was excellent, with a clear framework outlining the purpose and mechanics of TBL. Questions were promptly addressed, which was very reassuring.*”

### 4.4 Evaluation of TBL across all undergraduate year one courses

**FIGURE 3:**
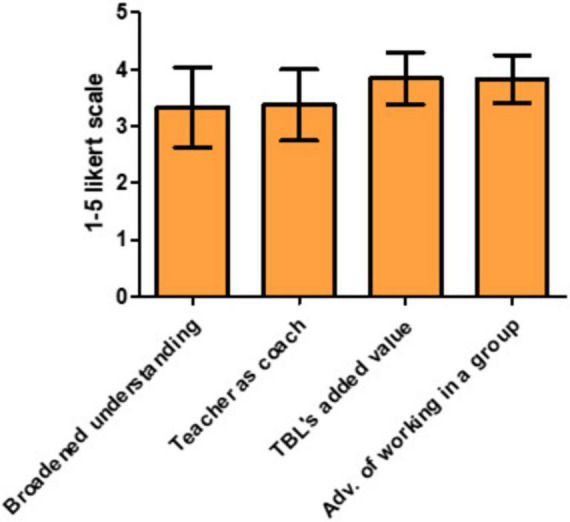
Scores on specific team-based learning (TBL) questions in the course evaluations. Results of the seven courses (average ± S.D.) are shown. Response per course was 35 ± 23.

To assess students’ perceptions of TBL, four specific questions were incorporated into the course evaluations of each course. The questions related to the effectiveness of TBL as a method for broadening understanding and the success of teachers in their coaching roles, received average scores of 3.5 [± 0.63 (SD, *n* = 7)] out of five. Questions assessing whether TBL was perceived as adding value and fostering collaborative work scored higher, at approximately 3.8 [± 0.42 (SD, *n* = 7)] out of five (Figure 3).

### 4.5 End-of-year survey for students

Students that participated in TBL at ACTA during the 2020–2021 academic year were invited to complete an evaluation questionnaire at the end of the year. During one of the last TBL sessions, a member of TBL team joined the online session and asked students to fill out the questionnaire via an anonymous link. Participation was voluntary and anonymous, with no identifying data collected. Students were informed that their feedback was crucial for improving TBL at ACTA. A total of 75 students out of 144 completed the questionnaire, and their responses were included in the analysis. The questionnaire received approval from the ACTA Ethical Committee (number 2021-82589).

The questionnaire was adapted from Parmalee et al. (17) and included 24 questions covering various aspects of TBL, including educational methods, teacher-role, organization, and group-work. Cronbach’s alpha was used to measure the internal consistency of the questions across the four categories. Seven questions addressed TBL in general (Cronbach’s alpha = 0.87), five questions focused on the teacher’s role during TBL sessions (Cronbach’s alpha = 0.80), nine questions were related to the organizational aspects of TBL (Cronbach’s alpha = 0.68) and three questions concerned team cooperation (Cronbach’s alpha = 0.87).

Questions were answered on a five-point Likert’s Scale, ranging from 1 = strongly disagree to 5 = strongly agree. Averages were calculated for items corresponding to the same scale. Table 1 presents response frequencies, means and standard deviations for each item. The table also shows means and standard deviations for each item, as well as mean composite scale scores.

**TABLE 1:** Results of a questionnaire for students on team-based learning (TBL) themes teaching method, the teacher, organization and collaborative work in groups.

	–	-	±	+	++	*n*	M	SD
**The following questions are related to teaching methods:**	**–**	**–**	**–**	**–**	**–**	**75**	**3.66**	**0.65**
I am better prepared for the application session thanks to the iRAT/tRAT.	1	1	19	47	7	75	3.77	0.69
The combination of the preparation day and the iRAT/tRAT have prepared me for the exam.	2	4	14	45	10	75	3.76	0.85
TBL sessions increase student participation.	1	5	14	43	12	75	3.80	0.84
Working in small groups has improved my understanding of the material.	2	0	17	49	7	75	3.79	0.72
TBL has helped me integrate various different concepts.	1	6	24	38	6	75	3.56	0.81
TBL sessions have taught me how to approach real dentistry related problems.	5	13	27	27	3	75	3.13	0.98
TBL is a useful addition to lectures and work groups.	4	4	11	37	19	75	3.84	1.04
**The following questions are related to the teacher**							**3.54**	**0.60**
The TBL teacher creates a safe environment in which I feel free to talk as a student.	0	2	23	37	10	75	3.69	0.79
The TBL teacher encourages me to contribute to the discussions.	0	7	21	40	7	75	3.63	0.79
The TBL teacher makes me enthusiastic to learn more about the subject.	0	13	28	30	4	75	3.33	0.83
The TBL teacher guides the discussions without passing judgment.	0	5	18	40	12	75	3.79	0.79
The TBL teacher has good time management.	0	13	33	24	5	75	3.28	0.83
**The following questions are related to organization**						**75**	**3.73**	**0.43**
It was clear what was expected of me in TBL.	1	0	16	51	7	75	3.84	0.64
I could easily find the information necessary for TBL.	0	2	17	46	10	75	3.85	0.67
I mostly had enough time for the iRAT.	0	0	3	33	39	75	4.48	0.58
I mostly had enough time for the tRAT.	0	1	2	33	39	75	4.47	0.62
I thought the mini-lecture after the tRAT was useful.	7	16	28	20	4	75	2.97	1.04
I thought that how the time was divided in the TBL application sessions was good.	2	25	18	28	2	75	3.04	0.97
I thought the online version of TBL was enjoyable.	2	4	11	38	20	75	3.93	0.94
The technical support for TBL went well (for example, entering and leaving breakout rooms).	0	4	9	46	16	75	3.99	0.74
I think the presence of the teacher in the breakout rooms is helpful.	4	9	24	25	3	75	3.05	0.99
**The following questions are related to collaboration in your team:**						**75**	**4.22**	**0.78**
I liked being in the same team for a whole year.	1	3	7	29	35	75	4.25	0.89
Throughout the year, we developed as a team.	1	2	9	27	36	75	4.27	0.88
I felt comfortable giving my team-members feedback through Feedback fruits.	0	4	12	28	31	75	4.15	0.88

Bold values indicate the average of the Likert -Scale scores of that category. 1–5 Likert’s Scale: –, firmly disagree to ++ firmly agree; *n*, number; M, mean; SD, standard deviation.

Students rated aspects of the educational method, the role of the teacher, and the organization of TBL between 3.54 and 3.73. The highest scores were for team collaboration, with an average of 4.22. When asked for additional feedback, many students suggested shortening the iRAT/tRAT phases, as they felt these phases involved unnecessary waiting.

Due to the COVID-19 pandemic, all TBL components were conducted online. At the end of the questionnaire, students were asked whether they preferred TBL in an online or face-to-face variant. The vast majority (77%) expressed a preference for continuing TBL online.

### 4.6 Dissemination

The principle of TBL and how it was implemented at ACTA was shared at a workshop during the Educational Day of the University of Amsterdam.

It is good to connect and to become a member of international TBL expert groups such as the Team-Based Learning Collaborative (TBLC). During the first year of implementation, Team TBL was invited by TBLC to give a presentation as part of the introductory series on how TBL was implemented at ACTA. The presentation, by Zoom, was viewed live, from every corner of the globe, stretching from the far-east to California.

## 5 Discussion on the practical implications, objectives and lessons learned

When we zoom out and put team-based learning and its objectives into a larger context, it is useful to go back to the pioneer of team-based learning, Larry Michaelson. Team-based learning was implemented in an era when larger audiences of students found their way to institutions of higher education. Faced with large student numbers, a search was made (and found!) for a method that could still serve large numbers of students in smaller groups (18). A quote from Michaelson that puts TBL’s mission in the broader perspective of academic learning: “Students need to learn and apply the power of reason gained through critical thinking before offering viewpoints and to apply this same approach when evaluating statements made by others. The extent to which a person accomplishes this process defines his or her competency in a given field.” (6). It is a method that helps in debating and argumentation of decisions, especially in medical science and dentistry, but also in related fields of study. Team-based learning has proven its value especially for the medical profession. Individuals from a curriculum with extensive TBL education had better long-term knowledge retention than students from more traditional curricula (19). When exposing one group of students to TBL while the other was not, and the groups switched halfway through the year, the students who initially received TBL and later transitioned to regular lessons appeared dissatisfied. The group that first received regular teaching and then TBL was happy with the enrichment that TBL provided (20). Notably, in the field of dentistry, the study by Jost et al. (21) demonstrated that TBL facilitates the process of clinical decision making. Their comparison of groups of students exposed to TBL versus those that were not, showed that the TBL group was better prepared and more capable of making clinical decisions.

**FIGURE 4:**
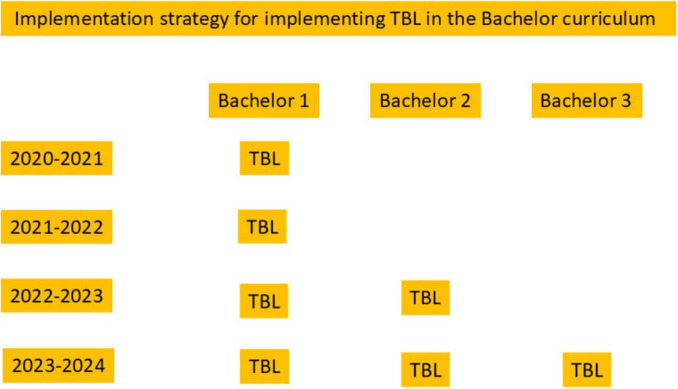
Implementation of team-based learning (TBL) at Academic Centre for Dentistry Amsterdam (ACTA). TBL was initiated in Bachelor-1 and paralleled the curricular reform of the bachelor phase from 2021 to 2022 onward. In essence, within 4 years, all bachelor courses contained a TBL-phase.

Team-based learning has been implemented as teaching method at ACTA for the past 4 years, initially delivered online for 2 years, followed by 2 years on-site. As the introduction of TBL coincided with a curricular reform, its implementation followed the reform’s phased rollout In other words, it was first introduced in all courses of Bachelor-1, the next year was a consolidation year, followed by introducing it in all Bachelor-2 courses and finally also in Bachelor-3 courses (Figure 4). The online version described here was replaced by a face-to-face variant in the third year. We can conclude that TBL has become an integral and sustainable part of the ACTA educational model. It has become part of the natural repertoire of teachers at ACTA, just as natural as giving a lecture in a large lecture hall and teaching in seminar format. Looking back at the implementation of TBL in the first year, part of its success is the step-wise and sustained approach of preparing teachers and students and of monitoring of the process through the first year(s). This seems pivotal for any successful and enduring curricular change (12), including team-based learning (9). Within ACTA, the TBL team was highly visible and both teachers and students found their way to Team TBL. It seems important to convey visibility and approachability and a willingness to advise and help.

With their first encounter of academic life, the first-year students had no other experience than online-teaching. And, although there are well-meant attempts to try and mimic a classroom setting using Zoom (22), one cannot but conclude that the Covid-19 pandemic has been a socially barren time that has had vast effects on the academic community that started in 2020. For instance, 1,400 American colleges transferred their on-site teaching to online within 1 month (23). Remarkably, the online version of TBL was considered positive in at least two respects. Students enjoyed their team, because it gave them some social anchorage point. Also, in the end-of-year survey, no less than 77% saw the online version of TBL as a positive example of online teaching that should be continued as an online version. Interestingly, a recently held survey amongst Bachelor-2 students at ACTA who had first experienced the online version and then 1 year with on-site TBL, responded entirely opposite to this, where the vast majority of students voted for on-site. Teachers even unanimously voted for on-site (unpublished results).

When looking back at how TBL was perceived by teachers, it was striking that the online Zoom environment allowed at a seemingly approachable way to invite teachers from other courses and team TBL to join such a session. Probably with any teaching method, there are teachers that fit-in quite naturally, whereas others find it difficult to adapt or do not see the benefits of using the new method. In that sense, when making it policy that every course has to offer at least one TBL session, the course coordinator should monitor which teachers are most suited for teaching TBL, where such a different, more coaching role is expected from teachers.

For the preparation phase of TBL, one should avoid overly complex pre-class materials which can overwhelm students (16), leading to low engagement or incomplete preparation. Although student engagement is influenced by many different factors, we observe that the complexity of pre-class materials plays an important role. As we know from motivation theory (24) and Bruner et al.’s (25) work on scaffolding, students thrive when the expected task complexity matches their abilities. Setting the complexity too low or too high can lead to demotivation and inadequate preparation for the TBL session. Therefore, we advise carefully considering the complexity of pre-class materials and ensuring that they are as well-suited as possible to the proficiency level of your students. Our initial instruction to teachers was to let the students prepare the course materials thus far. Students appreciated this, since the RAT phase could be seen as a light version of the exam, and gave them a fair idea to what extent they mastered the course work.

Based on recommendations by teachers, TBL application sessions are now held with two cohorts of 12 teams, rather than the initial four cohorts of six teams. Thus, there is less pressure on the teaching staff to teach four sessions. Another benefit is that the whole TBL (RAT phase and application session) can now be accommodated in 1 day. TBL has now been rolled-out and is part of all courses of the Bachelor’s program. The second and third year of the program is more and more focused on acquiring technical skills. Especially here, it was noticed that after TBL introduction, the test performance was higher than before TBL. However, also due to the installment of TBL at the same time as the online transition, it is difficult to gather clean data on before and after TBL.

When considering introduction of TBL, we would like to forward four tips for a successful incorporation.

### 5.1 Train faculty

Faculty must fully understand the principles and benefits of TBL to effectively facilitate sessions. Resistance to change from traditional methods can hinder implementation. Invest time in workshops, peer mentoring, and ongoing support of faculty to ensure all instructors are comfortable with TBL design and facilitation.

### 5.2 Engage students

Student engagement is critical to the success of TBL, as it depends on active participation inside but also outside the classroom. Engage students by explaining the benefits of TBL and align TBL carefully with final examination.

### 5.3 Align with curriculum goals

Team-based learning is most effective when integrated thoughtfully into a whole curriculum. Avoid fragmented learning experiences. Adapt the structure of your course and replace traditional lectures with a variety of pre-class preparation materials. Application exercises should promote active and relevant problem solving, decision-making and teamwork. Align the final exam with the topics and level of the application sessions, because examination drives learning and stimulates students to get the most out of the TBL sessions.

### 5.4 Secure administrative support

Institutional support ensures that sufficient time, resources, and infrastructure are available for TBL sessions, including appropriate classroom setups and technology.

## Data Availability

The raw data supporting the conclusions of this article will be made available by the authors, without undue reservation.
